# Cost-effectiveness of noninvasive telemedical interventional management in patients with heart failure: health economic analysis of the TIM-HF2 trial

**DOI:** 10.1007/s00392-021-01980-2

**Published:** 2021-12-11

**Authors:** Hanna Sydow, Sandra Prescher, Friedrich Koehler, Kerstin Koehler, Marc Dorenkamp, Sebastian Spethmann, Benjamin Westerhoff, Christoph J. Wagner, Sebastian Liersch, Herbert Rebscher, Stefanie Wobbe-Ribinski, Heike Rindfleisch, Falk Müller-Riemenschneider, Stefan N. Willich, Thomas Reinhold

**Affiliations:** 1 Division of Health Economics and Health Services Research, Institute of Social Medicine, Epidemiology and Health Economics, Charité-Universitätsmedizin Berlin, Luisenstr. 57, 10117 Berlin, Germany; 2 Centre for Cardiovascular Telemedicine, Medical Department, Division of Cardiology and Angiology, Charité-Universitätsmedizin Berlin, Berlin, Germany; 3 Department of Cardiology (Campus Virchow-Klinikum), Charité-Universitätsmedizin Berlin, Berlin, Germany; 4 Department of Cardiology and Angiology (Campus Charité Mitte), Charité-Universitätsmedizin Berlin, Berlin, Germany; 5 BARMER, Wuppertal, Germany; 6 AOK Nordost-Die Gesundheitskasse, Health Services Management, Berlin, Germany; 7 IGVresearch-Institut für Gesundheitsökonomie und Versorgungsforschung, Hamburg, Germany; 8 Faculty of Law, Business and Economics, University of Bayreuth, Bayreuth, Germany; 9 DAK Gesundheit, Health Services Research and Innovation, Hamburg, Germany; 10 Internal Medicine with Gastroenterology and Nephrology (CC 13), Charité-Universitätsmedizin Berlin, Berlin, Germany; 11 Saw Swee Hock School of Public Health, National University of Singapore, Singapore, Singapore

**Keywords:** Heart failure, Cost-effectiveness, Health economics, Telemedicine, Remote patient management

## Abstract

**Background:**

Noninvasive remote patient management (RPM) in patients with heart failure (HF) has been shown to reduce the days lost due to unplanned cardiovascular hospital admissions and all-cause mortality in the Telemedical Interventional Management in Heart Failure II trial (TIM-HF2). The health economic implications of these findings are the focus of the present analyses from the payer perspective.

**Methods and results:**

A total of 1538 participants of the TIM-HF2 randomized controlled trial were assigned to the RPM and Usual Care group. Health claims data were available for 1450 patients (*n* = 715 RPM group, *n* = 735 Usual Care group), which represents 94.3% of the original TIM-HF2 patient population, were linked to primary data from the study documentation and evaluated in terms of the health care cost, total cost (accounting for intervention costs), costs per day alive and out of hospital (DAOH), and cost per quality-adjusted life year (QALY). The average health care costs per patient year amounted to € 14,412 (95% CI 13,284–15,539) in the RPM group and € 17,537 (95% CI 16,179–18,894) in the UC group. RPM led to cost savings of € 3125 per patient year (*p* = 0.001). After including the intervention costs, a cost saving of € 1758 per patient year remained (*p* = 0.048).

**Conclusion:**

The additional noninvasive telemedical interventional management in patients with HF was cost-effective compared to standard care alone, since such intervention was associated with overall cost savings and superior clinical effectiveness.

**Graphical abstract:**

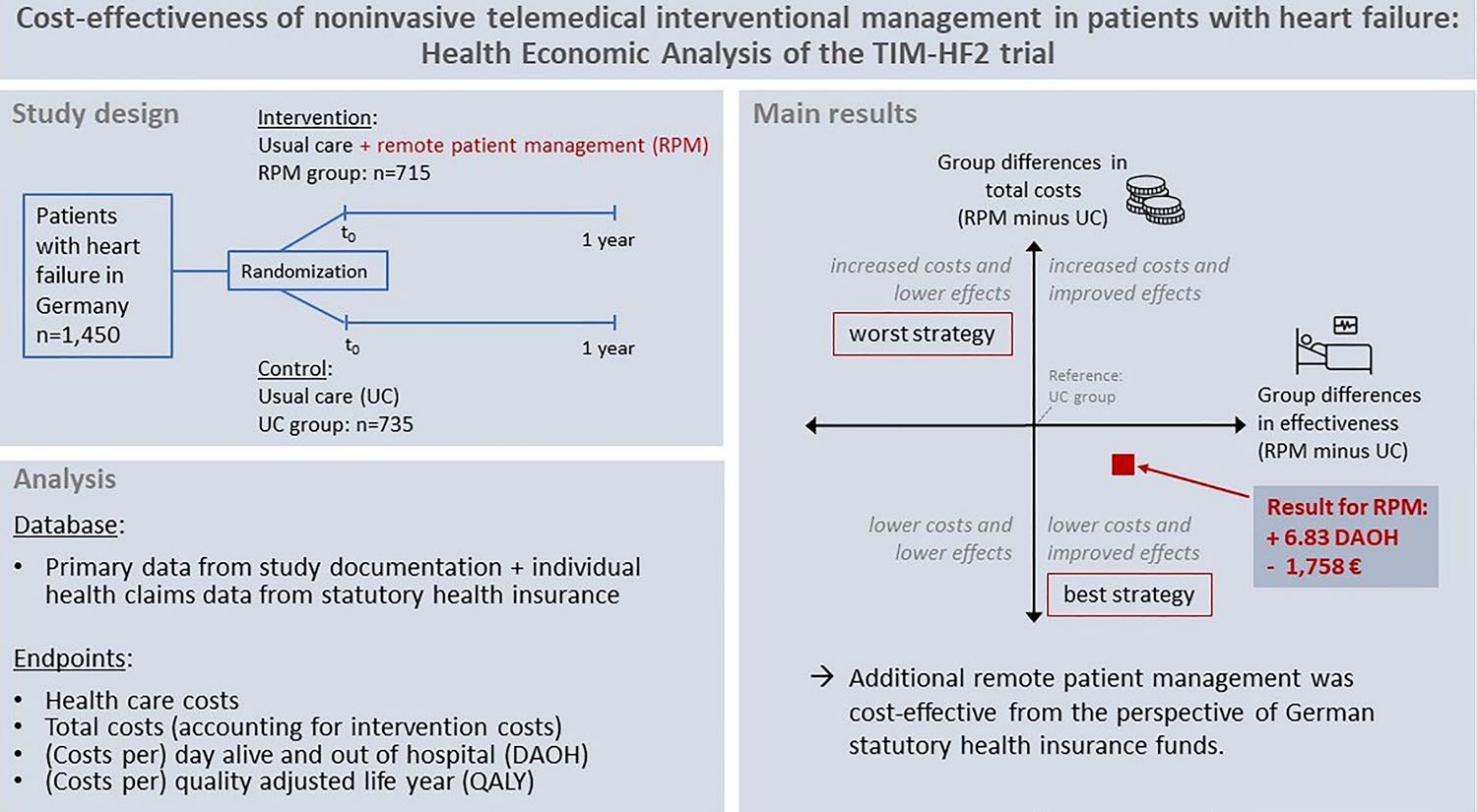

## Introduction

The prevalence of heart failure (HF) is estimated to be 3–4%, and it has an incidence of approximately 6 per 1000 persons per year [1, 2]. Approximately 456,012 patients had to be hospitalized for HF in Germany in 2018 [3]. For many years, HF has been one of the most common reasons for inpatient treatment in Germany, and there is still an increasing trend in the number of new cases each year. Approximately 1.6% (€ 5277 Mill.) of the total German health care expenditure was spent for the treatment of HF in 2015 [4].

As innovative therapies and new approaches to health care are required according to the high and rising relevance of HF, telemedicine is considered an important option [5]. Compared with face-to face medicine, telemedicine uses information technologies to overcome spatial and temporal distances between patients and physicians. Telemonitoring enables patients to be diagnosed and monitored remotely by those who are treating them and to react immediately. Different technologies can be used, from invasive approaches such as implantation of a physiologic sensor to noninvasive approaches. For the latter, patients usually receive specific medical devices for monitoring relevant vital parameters at home. To transmit the measured data to a telemedicine center or to the attending physician, information and communication technologies are used. Trained staff are able to detect critical changes in a patient’s health condition at an early stage and initiate the required interventions in time. Furthermore, telemedicine likely enhances the collaboration of different health care providers (e.g., hospitals, general practitioners, cardiologists, and nephrologists) to create an entire health database that enables an interdisciplinary, cross-sectorial, well-coordinated, and clearly structured therapy concept for each patient.

Numerous RCTs have examined the use of telemedicine in HF with different technologies and intensities of intervention. Several systematic reviews [6–9] were published, and they summarized the evidence about the effectiveness of telemedicine in HF. These reviews did not find consistently positive results, but some of the analysed intervention studies did show beneficial effects of telemedical interventions, especially on mortality and HF‐related hospitalizations.

Furthermore, the cost-effectiveness of noninvasive telemedical interventions has been investigated in several studies that have generated inconsistent evidence, with certain studies [10, 11] not showing any cost savings in favour of telemedical intervention compared to the controls. However, Vestergaard et al. [12] found that the Danish TeleCare North HF trial led to cost savings and was cost-effective in terms of health-related quality of life in favour of the intervention. Isaran et al. [13] studied remote patient monitoring in HF and chronic obstructive pulmonary disease (COPD), and found that the cost of emergency room visits and hospitalizations were reduced. For Germany, the CardioBBEAT trial investigated the cost-effectiveness of noninvasive telemonitoring as a primary endpoint. No incremental cost-effectiveness with respect to usual care was observed, although an improvement in quality of life in favour of the intervention group was found [14]. In addition to studies on noninvasive telemedical interventional management, the cost-effectiveness of invasive remote cardiac monitoring was also investigated. Based on data from the CHAMPION trial, the cost-effectiveness of a pulmonary artery pressure monitoring system was analysed. A simulation study [15] found that this intervention was cost-effective and presented an incremental cost-effectiveness ratio of €23,814 per quality-adjusted life year (QALY) gained in Germany.

The present analysis is based on the TIM-HF2 trial, which was designed to assess the impact of noninvasive structured remote patient management (RPM) on the morbidity and mortality in patients with HF in Germany. Koehler et al. [16] demonstrated that TIM-HF2 was effective in terms of reducing the percentage of days lost due to unplanned cardiovascular hospital admissions as well as all-cause mortality. The primary objective of the present health economic analysis was to assess the cost-effectiveness of telemedical interventional management in patients with HF based on the TIM-HF2 patient sample.

## Methods

The empirical health economic analysis was a prespecified secondary endpoint of the TIM-HF2 trial, and it used the clinical effectiveness data in combination with statutory health insurance (SHI) claims data provided by the participating patients’ SHI funds. The analysis is restricted to participating patients insured by one of the German statutory health insurance funds (*n* = 48) who had to obtain approval from their regulation authorities. Patients with private stand-alone health insurance were excluded due to considerable differences in reimbursement structures. Nevertheless, the share of patients with statutory health insurance in TIM-HF2 amounted to 94.7% of the entire study population.

### Study design and participants

The prospective, randomized, controlled, multicentre TIM-HF2 trial (ClinicalTrials.gov identifier: NCT01878630; DRKS-ID: DRKS00010239) included patients with New York Health Association (NYHA) class II or III HF, a history of HF hospitalization within 12 months prior to randomization, and either a reduced left-ventricular ejection fraction (LVEF) of ≤ 45% or with preserved LVEF of > 45% together with chronic therapy with oral diuretics. The main exclusion criteria were (1) haemodialysis, (2) major depression with a score higher than 9 on the PHQ-9 questionnaire at baseline, (3) hospitalization for HF within seven days prior to randomization, left-ventricular assist device, or coronary revascularisation and/or CRT implantation 28 days prior to randomization or scheduled coronary revascularisation, (4) transcatheter aortic valve implantation (TAVI), and (5) MitraClip and/or cardiac resynchronisation therapy (CRT) implantation 3 months after randomization.

Patients were randomized to either the RPM intervention in addition to usual care (RPM group) or to usual care only (UC group). The patients in the RPM group received four measuring devices for daily transmission: an ECG monitoring unit with a finger clip to measure oxygen saturation, a blood pressure monitor, a scale to measure body weight, and a tablet computer to record self-reported health status. All patient data were transferred automatically via a mobile phone network in an encrypted manner from the tablet computer to the Centre for Cardiovascular Telemedicine at Charité-Berlin (TMC), where a team of medical doctors and nurses was permanently available (24/7) to review the incoming data. Abnormalities in vital signs led to appropriate interventions (e.g., changing the patient’s medication, recommending an outpatient visit or inpatient treatment). Furthermore, the TMC provided an emergency call system and service. Further elements of the RPM intervention included an HF patient education programme initiated on the day of device installation followed by monthly patient telephone interviews and cooperation among the TMC, the patient’s general practitioner (GP), and cardiologist with respect to holistic patient management. The duration of the study was scheduled for 12 months, and the individual patient follow-up period extended up to 393 days after study onset for clinical endpoints.

The study complied with good clinical practice in accordance with the Declaration of Helsinki and the laws and regulations applicable in Germany. Written approval from the appropriate ethics committees was obtained. Patients provided written informed consent and granted permission for the TMC to contact their health insurance fund to receive health claims data. This process was approved by the German Federal Social Insurance Office and local authorities and performed for patients in both study groups.

Further details regarding the underlying study design, intervention, data collection, and primary clinical results have previously been reported by Koehler et al. [16, 17].

### Cost assessment

The health economic analyses were focused on resource consumption and costs from the perspective of German statutory health insurance. The total costs consist of the health care costs during the study period and the intervention costs for telemedical interventional management in the RPM group.

The health care costs during the study were calculated based on the costs for hospital treatment [total hospitalizations, unplanned cardiovascular (CV) hospitalizations, and unplanned HF hospitalizations], outpatient treatment, therapeutic appliances, health care products, rehabilitation treatment, medications, home nursing care, transportation costs, and sickness leave payments, which were billed at the expense of statutory health insurance for a disease-related work incapacity period longer than 6 weeks in Germany. These cost items were calculated based on the individual patient data provided by SHI funds.

Because the SHI data did not distinguish between planned and unplanned hospitalizations, the cost analysis used the hospitalization adjudications of the clinical endpoint committee, which was blinded to study group assignment, adjudicated all hospitalizations using prospectively defined criteria. A hospitalization was considered as cardiovascular, if the admission reason was clearly associated with a deterioration of a cardiovascular condition (e.g., newly occurred or recurrent arrhythmia, acute coronary syndrome, myocardial infarction, worsening of heart failure, myocarditis, and endocarditis) or with the need of inpatient cardiological diagnostics. Additionally, heart failure hospitalizations were examined, which were defined as a subcategory of cardiovascular hospitalizations. Unplanned hospitalizations were characterized by an occurrence of new symptoms and/or worsening of existing symptoms with the need for immediate admission to a hospital for intensified diagnostics and therapy, while planned hospitalizations were those for diagnostic procedures, elective interventions (like a device battery change), or planned operations. Further information on these criteria were published in the clinical endpoint committee charter (see Koehler et al., Appendix S2 [17]).

The RPM intervention costs were solely observed in the RPM group. The intervention costs mainly included the costs for technical infrastructure (6%) and patients measuring devices (16%) and personnel costs for running the TMC Berlin (78%). The share of personnel costs appears to be high, which is based on the 24/7 accessibility of the TMC. The average annual intervention costs per participant in the RPM group were estimated at € 1413.54.

### Effectiveness measures

To estimate the cost-effectiveness of telemedical interventional management, the individual days alive and out of hospital (DAOH) during the study period was predefined as the primary effectiveness measure for the present health economic analysis. This information was derived from SHI claims data. Information on mortality was obtained from the primary study documentation.

According to common international health economic cost-effectiveness literature, quality-adjusted life years (QALYs) were defined as a further secondary effectiveness measure. The calculation of QALYs was based on the results of the German EQ-5D-3L questionnaire, a generic instrument for measuring health-related quality of life [18]. Patients were asked to complete this questionnaire at baseline and at the end of the study. The resulting health state utility indices for each time point were used for the QALY measurements. Therefore, the area under the curve was calculated individually, with linear changes assumed between both longitudinal utility values [19]. QALYs could only be calculated if both the baseline value and the last value were available, except for deceased patients. For participants who died during the study follow-up, a health state utility of 0 (zero) was assumed for the date of death, which was considered in the QALY calculation.

### Cost-effectiveness

For the cost-effectiveness analyses, the results for the total costs and effectiveness were combined separately. The incremental cost-effectiveness ratio (ICER) was only calculated in case of additional total costs and superior effects in terms of the DAOH or QALY for the RPM group, and it showed the additional costs for reaching one additional effectiveness unit gained. Other outcome constellations were assessed qualitatively and by calculating the cost per outcome unit to obtain information on the cost-effectiveness.

### Statistical analysis

Data were analysed based on the intention-to-treat (ITT) approach. Since costs were investigated from an SHI perspective, only patients with SHI claims data were included. To account for individual variations in the study duration, the costs were weighted with the study duration to derive the total cost per year. If patients had died during the study period, no weighting of costs was applied. Since the share of missing values was smaller than 2% for every single cost category and equally distributed between the RPM group and the UC group, missing values were not replaced. Baseline characteristics are presented as proportions (%) or mean values with standard deviations (SD). The results are described as the means, standard deviations (SD), and 95% confidence intervals (95% CIs). Mean costs were presented in two ways. First, the mean values are reported in relation to the total population (including those who had no costs in this area), and second, the means are presented relative to those patients who are actually affected by resource consumption. Since the distribution of health care costs is usually very skewed, a normal distribution assumption is violated. To consider the skewness of the cost data, a generalized linear model with gamma distribution and log-link function [20, 21] was used to estimate the mean costs, 95% confidence intervals (95% CIs), and mean group differences between the RPM and usual care group. Effectiveness measures were compared using Student’s t test. No adjustment for multiple testing was conducted [22]. For all statistical analyses, SPSS version 27 (IBM Inc.) was used. All results were verified using R version 4.0.3.

### Sensitivity analysis

Sensitivity analyses were conducted to evaluate the level of confidence that may be associated with the results and conclusion of the cost analysis. Uncertainties in the cost analysis were mainly associated with two factors: the assumption of weighting by the individual study duration and the heterogeneity of the patient sample. To determine whether the results are sensitive to the weighting procedure, the costs of the deceased patients were also weighted by their individual duration of study participation. Additionally, a probabilistic sensitivity analysis was used to account for heterogeneity of the study population in terms of health care resource consumption and effectiveness. Therefore, a bootstrap analysis of the original population sample with 1000 random resamplings with replacement was performed to assess the extent to which the results may change. The results were plotted into a cost-effectiveness plane to obtain a graphical overview of the variance of our results.

## Results

### Study sample and baseline characteristics

**Fig. 1:**
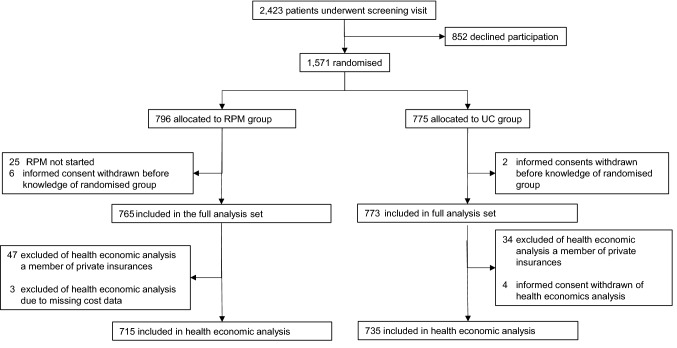
Analysis flowchart

From the original study sample (full analysis set) of *n* = 1538 patients (*n* = 765 RPM group, *n* = 773 UC group), a total of *n* = 1450 patients (94.3%) were considered based on SHI claims data from 48 different SHIs in the present health economic analysis (*n* = 715 RPM group 93.5%, *n* = 735 UC group 95.1%). For further details, see Fig. 1. The patient baseline characteristics were well balanced between both groups (Table 1), and compared to the original slightly larger study sample, no relevant differences were observed. The mean age of the patients at study onset was 70.5 years (SD 10), and 69% were male. Both groups had comparable distributions of cardiovascular risk factors, such as smoking status, hyperlipidaemia, diabetes, and NYHA classes. The actual mean individual study duration was 11.6 months in the RPM group and 11.7 months in the UC group.

**Table 1:** Baseline characteristics

	RPM group (*n* = 715)		UC group (*n* = 735)		Group differences (RPM minus UC)
Sex					
Male	492	68.8%	504	68.6%	Δ pp 0.2
Female	223	31.2%	231	31.4%	Δ pp − 0.2
Living in an urban area vs. rural area					
Rural	431	60.3%	437	59.5%	Δ pp 0.8
Urban	284	39.7%	298	40.5%	Δ pp − 0.8
Hyperlipidaemia					
No	283	39.6%	303	41.3%	Δ pp − 1.7
Yes	393	55.0%	395	53.8%	Δ pp 1.2
Unknown	39	5.5%	36	4.9%	Δ pp 0.6
Smoking status					
Non-smoker	353	49.4%	367	49.9%	Δ pp − 0.5
Former smoker	266	37.2%	291	39.6%	Δ pp − 2.4
Smoker	75	10.5%	54	7.3%	Δ pp 3.2
Unknown	21	2.9%	23	3.1%	Δ pp − 0.2
Diabetes mellitus					
No	388	54.3%	392	53.3%	Δ pp 1.0
Yes	327	45.7%	343	46.7%	Δ pp − 1.0
NYHA class					
I	2	0.3%	6	0.8%	Δ pp − 0.5
II	364	50.9%	374	50.9%	Δ pp 0.0
III	347	48.5%	353	48.0%	Δ pp 0.5
IV	2	0.3%	2	0.3%	Δ pp 0.0

	Mean	SD	Mean	SD	
Age	70.5	10.5	70.5	10.5	Δ mean 0.0
BMI [kg/m^2^]	29.8	6.4	29.8	6.1	Δ mean 0.0
Bodyweight (kg)	87.2	20.3	87.4	19.7	Δ mean − 0.2
Blood pressure (mm HG)					
Systolic	125.7	18.7	125.3	19.9	Δ mean 0.4
Diastolic	73.9	11.3	73.8	11.4	Δ mean 0.1
Pulse (beats per min)	72.6	13.8	72.1	13.9	Δ mean 0.5
LVEF	41.4	13.4	40.7	13.5	Δ mean 0.7
Primary cause of heart failure					
Ischaemic cause (coronary artery disease or myocardial infarction)	284	39.7%	308	41.9%	Δ pp − 2.2
Hypertension	122	17.1%	139	18.9%	Δ pp − 1.8
Dilated cardiomyopathy	165	23.1%	164	22.3%	Δ pp 0.8
Other	144	20.1%	124	16.9%	Δ pp 3.2
Medical history					
Coronary revascularisation (PCI)	241	33.8%	281	38.2%	Δ pp − 4.4
Coronary artery bypass surgery	128	17.9%	138	18.8%	Δ pp − 0.9
TAVI	22	3.1%	26	3.5%	Δ pp − 0.4
Mitral clip	25	3.5%	33	4.5%	Δ pp − 1.0
Cardiac surgery for valves	82	11.5%	64	8.7%	Δ pp 2.8
Implantable cardioverter defibrillator	199	27.8%	224	30.5%	Δ pp − 2.7
Cardiac resynchronisation therapy	105	14.7%	117	15.9%	Δ pp − 1.2
Ablation of pulmonary veins	65	9.1%	49	6.7%	Δ pp 2.4
Concomitant treatment					
ACE inhibitors or ARBs	584	81.7%	612	83.3%	Δ pp − 1.6
ARN inhibitors	42	5.9%	44	6.0%	Δ pp − 0.1
β blockers	659	92.2%	676	92.0%	Δ pp 0.2
Aldosterone antagonists	408	57.1%	392	53.3%	Δ pp 3.8
Loop diuretics	674	94.3%	687	93.5%	Δ pp 0.8
Thiazides	180	25.2%	178	24.2%	Δ pp 1.0
Other diuretics	4	0.6%	1	0.1%	Δ pp 0.5
Vitamin K antagonists	257	35.9%	263	35.8%	Δ pp 0.1
Antiplatelet therapy	95	13.3%	120	16.3%	Δ pp − 3.0
NOACs	189	26.4%	194	26.4%	Δ pp 0.0
Lipid-lowering drugs	432	60.4%	432	58.8%	Δ pp 1.6
Insulin	162	22.7%	166	22.6%	Δ pp 0.1
Oral hypoglycaemic drugs	194	27.1%	178	24.2%	Δ pp 2.9
Ivabradine	21	2.9%	40	5.4%	Δ pp − 2.5
Calcium antagonists	157	22.0%	178	24.2%	Δ pp − 2.2
Nitrates	36	5.0%	47	6.4%	Δ pp − 1.4
Digitalis glycosides	110	15.4%	126	17.1%	Δ pp − 1.7
Antiarrhythmic drugs	90	12.6%	92	12.5%	Δ pp 0.1

Laboratory measurements	Median	IQR	Median	IQR	
Haemoglobin (mmol/L)	8	(7–9)	8	(8–9)	Δ median 0
Serum sodium (mmol/L)	140	(137–142)	140	(138–142)	Δ median 0
Potassium (mmol/L)	5	(4–5)	5	(4–5)	Δ median 0
Serum creatinine (µmol/L)	109	(87–143	110	(88–149)	Δ median − 1
Estimated GFR (mL/min per 1.73 m^2^ of body surface area, Cockcroft-Gault)	62	(44–89)	62	(45–87)	Δ median 0
NT-proBNP (pg/mL)	1394	(622–3184)	1491	(594–3085)	Δ median − 97
MR-proADM (nmol/L)	1	(0.8–1.5)	1	(0.8–1.5)	Δ median 0

*pp* percentage points, *SD* standard deviation, *IQR* interquartile range

### Cost results

From the SHI perspective, patients in the RPM group had lower health care costs than patients in the UC group (Table 2). The average total health care costs amounted to € 14,412 in the RPM group and € 17,537 in the UC group. A mean cost difference of € 3125 per patient year in favour of the RPM group was observed, which is economically relevant and statistically significant (*p* = 0.001). Group cost savings favouring the RPM group tended to be lower for those patients with preserved LVEF (≥ 50%) compared to those with reduced (≤ 40%) or mid-range LVEF (41–49%). Even after including the intervention costs, the total cost of the RPM group (mean: € 15,779) remained lower than the total cost of the UC group (mean: € 17,537). The resulting saving of € 1758 for the RPM group was statistically significant, as well (*p* = 0.048).

**Table 2:** Health care costs and total costs (in €) of the intervention per patient year with confidence intervals and p values using gamma regression

	RPM group (*n*=715)				UC group (*n* = 735)				Mean difference (RPM minus UC)	*p* value
	*n*	Estimated mean	Asymptotic lower 95% CI	Asymptotic upper 95% CI	*n*	Estimated mean	Asymptotic lower 95% CI	Asymptotic upper 95% CI		
Hospital										
All cases	715	8328.92	6727.16	9930.67	735	10,816.99	8765.25	12,868.74	− 2488.07	0.058
Only cases with costs	436	13,658.64	12,245.92	15,234.35	477	16,660.99	15,009.61	18,494.06	− 3002.34	0.010
Unplanned CV hospitalisations										
All cases	715	3921.68	2990.70	4852.66	735	5693.63	4360.52	7026.74	− 1771.95	0.029
Only cases with costs	232	12,086.19	10,512.60	13,895.33	252	16,606.40	14,526.12	18,984.60	− 4520.21	0.001
Unplanned HF hospitalisations										
All cases	715	2256.25	1692.45	2820.06	735	3219.81	2426.25	4013.38	− 963.56	0.047
Only cases with costs	130	12,409.34	10,350.95	14,877.06	183	12,931.99	11,098.80	15,067.97	− 522.65	0.733
Outpatient										
All cases	708	1345.86	1257.22	1434.50	729	1441.11	1343.25	1538.96	− 95.25	0.147
Only cases with costs	699	1363.18	1284.43	1446.75	725	1449.06	1366.82	1536.24	− 85.88	0.151
Medication										
All cases	712	2700.64	2511.90	2889.38	732	2788.98	2596.75	2981.21	− 88.34	0.520
Only cases with costs	708	2715.89	2547.24	2895.70	724	2819.79	2646.58	3004.34	− 103.90	0.414
Rehabilitation										
All cases	701	107.84	81.80	133.88	716	193.42	147.21	239.62	− 85.58	0.001
Only cases with costs	43	1757.86	1294.72	2386.66	64	2163.75	1684.02	2780.13	− 405.89	0.298
Therapeutic appliances										
All cases	715	180.40	144.16	216.65	735	197.31	158.21	236.40	− 16.90	0.534
Only cases with costs	277	465.64	409.88	528.98	294	493.24	435.80	558.25	− 27.60	0.525
Health care products										
All cases	713	541.86	437.46	646.26	733	711.98	576.68	847.27	− 170.12	0.048
Only cases with costs	375	1030.23	897.34	1182.81	414	1260.56	1105.30	1437.63	− 230.33	0.039
Home nursing										
All cases	707	250.59	189.63	311.55	725	415.25	315.50	515.01	− 164.67	0.004
Only cases with costs	82	2160.46	1611.86	2895.79	101	2980.70	2289.22	3881.05	− 820.24	0.111
Transportation										
All cases	707	676.50	535.45	817.55	725	660.88	524.80	796.95	15.62	0.876
Only cases with costs	299	1599.60	1391.48	1838.84	322	1487.98	1300.96	1701.87	111.62	0.465
Sickness leave payments										
All cases	703	488.56	363.75	613.38	725	296.29	221.76	370.83	192.27	0.006
Only cases with costs	41	8376.91	6251.18	11,225.48	28	7671.61	5383.44	10,932.34	705.30	0.706
Total health care costs	688	14,411.81	13,284.30	15,539.32	703	17,536.52	16,179.27	18,893.78	− 3124.72	0.001
Intervention costs	715	1366.37	1352.13	1380.62	735	–	–	–	1364.42	
Total costs (intervention costs + total health care costs)	688	15,778.72	14,649.68	16,994.77	703	17,536.52	16,179.27	18,893.78	− 1757.81	0.048

Hospital costs are the largest cost category from the perspective of statutory health insurance, and this was also observed in this study. Patients in the RPM group had an average hospital cost of € 8329 per patient year, and patients in the UC group had an average hospital cost of € 10,817 per patient year. Inferentially, the RPM group had a lower hospital cost by € 2488 per patient year (*p* = 0.058) than the UC group. However, hospital costs were also lower when only hospital cases were considered. The resulting cost difference in this case was € 3002 (*p* = 0.010), showing that the RPM group not only had lower costs due to more zero-cost cases but also had lower costs in the case of hospital treatment than the UC group. A large difference of € 1772 for the costs of unplanned CV hospitalizations was observed in favour of the RPM group. The costs of unplanned CV hospitalizations differed significantly (*p* = 0.029) between the groups. Considering only the cases with unplanned CV hospitalizations, the mean difference of costs is notably higher (€ 4520) for the UC group (*p* = 0.001). Regarding the costs of unplanned HF hospitalizations, the mean difference between the groups amounted to € 964 for all cases, which significantly (*p* = 0.047) favours the RPM group. Similarly, for cases of unplanned HF hospitalizations, a mean difference of € 523 in favour of the RPM group was observed, although this result was not statistically significant (*p* = 0.733).

The costs of outpatient treatment were not significantly different in the RPM group compared to the UC group (€1346 vs. €1441, *p* = 0.147).

Medication costs per patient year amounted to € 2701 in the RPM group and € 2789 in the UC group.

For inpatient and outpatient rehabilitation costs, a significant but rather small cost difference of € 86 (*p* = 0.001) in favour of the RPM was observed. Considering only the cases with rehabilitation, it is apparent that the cost difference € 406 (*p* = 0.298) was larger, but the proportion of patients with rehabilitation was low in both groups, with a slightly higher amount in the RPM group (RPM 6.1%; UC 8.9%).

The costs of therapeutic appliances consist mainly of the cost of physiotherapy, occupational therapy, and speech therapy. Both groups had very similar costs of therapeutic appliances (RPM group: € 180; UC group: € 197; difference: €17, *p* = 0.534). The same result was found when only cases that received therapeutic appliances were considered.

Another cost category is health care products, which include a wide range of different medical aids, such as visual, hearing, and orthopaedic aids, as well as technical products and devices. The costs of health care products amounted to € 542 in the RPM group and € 712 in the UC group (difference: € 170, *p* = 0.048). When only the cost cases were compared, the cost difference was slightly higher at € 230 and remained significant (*p* = 0.039).

The RPM group induced costs of € 251 for home nursing, while the UC group induced costs of € 415, for a difference of € 164 (*p* = 0.004). Only 11.5% of the patients in the RPM group and 13.9% of patients in the UC group received home nursing. In these patients, a substantially higher but statistically insignificant cost difference of € 820 (*p* = 0.111) in favour of the RPM group was observed.

Transport costs included the costs for ambulance services and patient transports. The cost amounted to € 676 in the RPM group and € 661 in the UC group. The cost difference was not statistically significant (*p* = 0.876). Forty-two percent of the RPM group and 44% of the UC group used medical transportation services. In this subpopulation, the RPM group had a € 112 (*p* = 0.465) higher cost than the UC group, which resulted from a higher proportion of expensive transport services (for example, by ambulance helicopter) in the RPM group.

The last cost category consisted of sickness leave payments, which were covered by statutory health insurance from the 7th week of the patient’s incapacity for work and were dependent on the individually earned income. In the RPM and UC group, sickness leave payments of € 489 and € 296 were found, respectively, which resulted in a mean difference of € 193 (*p* = 0.006) in favour of the UC group. However, only a few patients received sickness leave payments (RPM group: 5.8%; UC group: 3.9%), because more than half of the participants were already retired.

### Effectiveness

With regard to the effectiveness measure of this health economic analysis, an improvement in DAOH and QALY was observed. Compared to the UC group, patients in the RPM group experienced more days alive and out of hospital per patient year (RPM group mean DAOH of 339.08 (95% CI 334.66–343.5) vs. UC group mean DAOH of 332.25 (95% CI 327.35–337.14), mean DAOH difference 6.83, *p* = 0.045). For the QALY values, a nonsignificant difference of 0.13 was found (RPM group mean QALY of 0.709 (95% CI 0.690–0.728) vs. UC group mean QALY of 0.696 (95% CI 0.677–0.715), *p* = 0.347). These results are in line with the results of the Minnesota Living for Heart Failure Questionnaire already published by Koehler et al. [16].

### Cost-effectiveness

After combining the cost and the effectiveness results, the overall treatment of the RPM group was associated with cost savings of € 1761 and superior clinical effectiveness relative to the UC group. Dividing the total cost by the total number of DAOH per group, the cost per DAOH was lower in the RPM group (€ 45) than the UC group (€ 50). The same applies to the cost per QALY, with € 22,741 for the RPM group and € 25,890 for the UC group. In light of this result, it is not indicated to calculate the incremental cost-effectiveness ratio (see “Methods”).

**Fig. 2:**
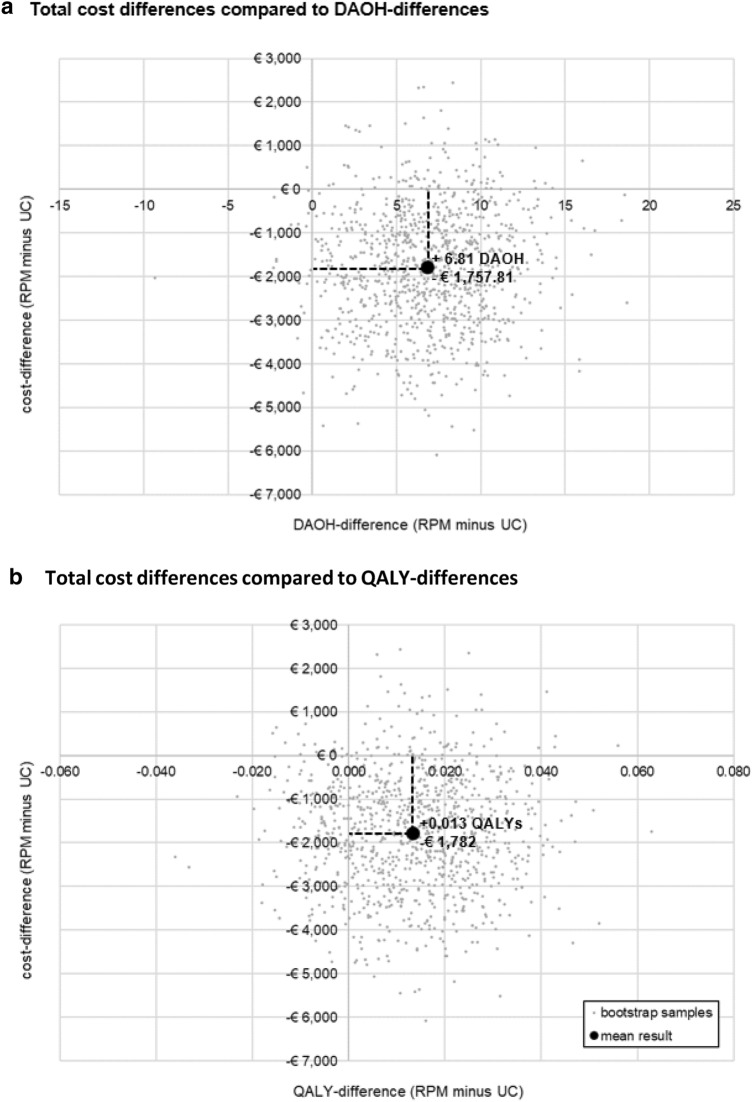
Bootstrap-based sensitivity analyses

These results proved to be robust in the sensitivity analyses. Weighting by individual study duration demonstrated that the mean differences in the total costs were comparable with the results of the base-case analysis and demonstrated the advantage of RPM. Furthermore, bootstrap analyses showed that the majority of resampled results were found within the lower down quadrant (89.5% for cost/DAOH, 76.4% for cost/QALY), thus showing lower total costs and superior effectiveness, particularly for the effect on DAOH (Fig. 2).

## Discussion

### Key results

From the statutory health insurerance perspective, the RPM group had significantly lower health care costs per patient year than the UC group, even if the intervention costs were included. The present health economic analysis is also largely confirmed superior effectiveness with regard to primary clinical endpoints that were previously published [16].

Observed cost savings were mainly driven by significantly lower hospital costs in the RPM group, primarily as a result of the lower share of unplanned CV and HF-related hospitalizations in the RPM group compared to the UC group. This finding is consistent with the main clinical findings of TIM-HF2 reported by Koehler et al. [16] and the main objective of the trial, which was to reduce unplanned cardiovascular hospitalization and all-cause mortality. A relation can also be seen with regard to home nursing costs, which were significantly lower in the RPM group. Home nursing is often provided after discharge from the hospital to shorten hospital stays or even to avoid hospitalization. Since hospitalizations were less frequent in the RPM group, it is conceivable that the costs and the utilization of home nursing were also lower due to the intervention. In contrast, we did not observe a meaningful effect of the intervention on outpatient costs, which can be explained by the fact that nearly all HF patients required regular outpatient monitoring and only very few patients without documented outpatient treatment were found in the insurance data. In addition, outpatient doctors’ visits in Germany are remunerated with flat rates in many cases; therefore, possible differences in service utilization between the groups are not necessarily reflected in the costs. According to standard pharmacological therapy after a diagnosis of HF, we did not find differences in medication costs. Similarly, cost differences for therapeutic appliances were not observed.

Our findings are mainly in line with the results of a previous study from Denmark [12] that demonstrated a reduction in total health care costs from the public payer perspective for a noninvasive telemonitoring intervention in HF. Studies such as Blum et al. [10], which found no cost saving effects of noninvasive telemedicine in HF, were mostly older and had a comparatively small number of participants. The scope and mode of intervention also varied between studies. In particular, it was notable that not all interventions provided a 24/7 accessibility. Especially, an emergency service was offered only in our study. The effect of this service was already investigated by Winkler et al. [23], who showed that the total number of unplanned cardiovascular hospitalizations can be reduced by 24/7 remote patient management due to a reduction in emergency visits following emergency calls to the TMC. For an invasive telemonitoring intervention, cost-effectiveness but no cost savings were observed, which might be the consequence of the high intervention costs and potentially associated complications of an implantable device [15]. However, when comparing our study with those already published, it must be considered that most of the studies originate from health care systems that are structurally different from the German system. Due to differences of reimbursement systems between the countries, comparability in terms of costs is therefore limited [24].

### Strengths

The present analysis has some valuable characteristics and strengths. One of the leading strengths is the fact that in addition to a large sample size in the TIM-HF2 trial, SHI claims data for cost analysis were obtained for 94.7% of participants from 48 different German statutory health insurances. Thus, biases due to specific characteristics in the population of one single statutory health insurance do not exist. Due to the nature of SHI claims data, cost information was highly complete, and only approximately 4% of our sample could not be included in the calculation of total costs caused by missing values in one or more cost categories. The almost complete availability of cost data for the study population could only be achieved, because the health economic analyses were planned during the initial study development phase and data deliveries were successively monitored. Another important strength of this study lies in the multicentre nature of the study, with participants from all over Germany, including both rural and urban regions. Our findings can therefore be seen as applicable to Germany as a whole.

### Limitations

Nevertheless, the analysis contains potential limitations. Health care costs from the perspective of statutory health insurance depend on the administered prices of the reimbursement systems in Germany, and these prices are not comparable with market prices. For this reason, for many of the cost categories (e.g., medication costs and nursing home care costs), it was impossible to draw conclusions from the amount of costs to the quantity and scope of the utilized services.

Therefore, the transferability of our findings to other health care systems is limited, as national health systems differ structurally in many aspects by country. Furthermore, 5.3% of the study population had private health insurance and was therefore excluded from the analysis due to different reimbursement systems between private and statutory health insurance. Consequently, it is not possible to draw conclusions about the costs of the entire study population.

Even though completed data were received for health care utilization periods that overlapped the study period from most statutory health insurance but not all, it is possible that certain cost parameters (e.g., hospitalization) may be slightly underestimated. However, since the study participants were randomly distributed with respect to their statutory health insurance company, the results are not expected to be biased. The value of the QALY calculation was reduced, since quality of life (EQ-5D) was only assessed at two measurement points (beginning and follow-up). Although the study also used the Minnesota Living for Heart Failure Questionnaire to measure the disease-specific quality of life, in further studies, a larger number of measurement points and a more specific generic questionnaire would be useful to more accurately determine the overall quality of life. Since the underlying TIM-HF2 trial was performed to identify the primary clinical outcome and the present secondary analysis was not adjusted for multiple testing, the reader should keep in mind that the significant results for secondary outcomes presented are only explorative and used to generate hypotheses.

### Outlook

Remote patient management for heart failure patients with noninvasive devices was published as a medical service in the Federal Gazette on March 30, 2021, and the procedures, costs, and quality standards will be defined by the Federal Joint committee (G-BA). In the future, technological progress (e.g., application of artificial intelligence) and interventions offered to a larger population could realize economies of scale, which might lead to a reduction in intervention costs and thus have an even higher potential for cost savings and associated cost-effectiveness of RPM. However, costs are driven by personnel costs, and relevant cost savings could especially be achieved by the integration of artificial intelligence in the preprioritisation of a high number of patients or networking of telemedical centers for higher workloads outside business hours [25, 26].

## Conclusion

In Germany, additional noninvasive telemedical interventional management in patients with heart failure was associated with overall cost savings for statutory health insurance and superior effectiveness in terms of lower mortality and a higher number of days without hospitalization due to CV and HF. According to these results, the investigated noninvasive telemedical interventional management was found to be cost-effective. These health economic results are robust, particularly due to the high level of completeness of the underlying cost data provided by participating statutory health insurance funds for the study participants.

## Data Availability

To fulfil the legal requirements to obtain health claims data in Germany, researchers must obtain permission for a specific research question from the German Federal Office for Social Security. Additionally, researchers must conclude a contract with statutory health insurance regarding data access. Moreover, a request has to be approved by the data protection officer at both the statutory health insurance and the research institute as well as the local ethics committee.
